# Editor’s introduction

**DOI:** 10.1186/s40854-023-00480-8

**Published:** 2023-03-23

**Authors:** Gang Kou

**Affiliations:** Southwestern University of Finance and Economics, Chengdu, China

The 38th issue of Financial Innovation (FIN), Volume 9, No. 2 (2023) presents 23 papers contributed by authors and co-authors from nineteen countries and areas: Canada, Chile, China, France, Germany, Italy, Netherlands, New Zealand, Oman, Portugal, Romania, Russia, Spain, South Africa, Taiwan, Türkiye, UK, USA and Vietnam. These papers can mainly be categorized into three sub themes.

**• Financial risk management and analysis**

Gómez-Águila et al. (2022) introduce a new procedure to improve the estimation of the Hurst exponent in financial time series and prove that pure price series are not necessarily self-similar. Yeh and Liu (2023) use visualization techniques to help explore the Growth Value Model (GVM) intuitively. De Blasis (2023) addresses the calibration issues of the weighted-indexed semi-Markov chain (WISMC) model applied to high-frequency financial data. Ivanyuk (2023) presents an optimization approach—residual-based bootstrap averaging (RBBA) and shows why and how it works. Monteiro et al. (2023) examine the linear interdependencies between international industries, with a focus on the relationships between the US and other six countries, focusing mainly on international stock indexes or firm-level returns. Mo et al. (2023) present an adaptive measurement model of spatiotemporal correlation coefficients based on the clustering theory and conduct an empirical analysis based on the model. Loughran and McDonald (2023) find a slight positive correlation between industry performance and the use of pandemic-related words, as opposed to the expected negative correlation. Gupta and Pierdzioch (2023) prove that aggregate and state-level economic conditions can predict the subsequent realized volatility of oil price returns.

**• Behavior finance**

Cheng et al. (2023) find that rumor propagation outperforms management shocks and other variables in predicting abnormal trading behavior. Wang and Lee (2023) chose three life insurance stock futures in India and one in Taiwan as samples and examine that insurance futures are a safe haven for COVID-19 pandemic risks. Duréndez et al. (2023) find that Management control systems (MCS) and managers’ risk attitudes fully mediate the relationship between financial literacy (FL) and innovation. Alp et al. (2023).examine the presence of investor overconfidence by employing an artificial intelligence technique and a nonlinear approach to impulse responses to analyze the impact of different return regimes on the overconfidence attitude. Madeira (2023) demonstrates how households’ characteristics affect their choice of lenders, consumer debt amounts and default behavior. Cruz et al. (2023) propose an approach to the profile of microcredit holders from the Hispanic minority in the US to identify the factors that explain the punctuality of their microcredit repayments. Zhou et al. (2023) develop a behavioral heterogeneous agent model (HAM) comprising fundamentalists, chartists, and stabilizers to investigate investors’ dynamic switching mechanisms under government intervention.

**• Stock market**

Htun et al. (2023) find that correlation criteria, random forest, principal component analysis, and autoencoder are the most widely used feature selection and extraction techniques with the best prediction accuracy for various stock market applications. Zhang et al. (2023) propose three novel strategies to address common issues in predicting high-frequency stock prices using machine learning methods. Cepoi et al. (2023) explore the impact of the COVID-19 pandemic on information asymmetry for the case of the Romanian capital market. Meng et al. (2023) examine the effect of stock market liberalization on market efficiency by using a recent stock market liberalization reform policy in China—the Stock Connect—as a quasi-natural experiment. Mensi et al. (2023) provide strong evidence of quantile dependence between gold and stock returns. Li and Chen (2023) conduct a theoretical analysis of low-price collusion in SBIC from the perspective of strategic interaction between institutional investors and the regulator. Mestre (2023) proposes a wavelets approach to estimating time–frequency-varying betas in the capital asset pricing model (CAPM) framework. Carrillo-Hidalgo et al. (2023) develop a dynamic regression model to predict the behavior of shares in the Spanish tourism sector according to the evolution of the COVID-19 pandemic in the medium term. It is confirmed that both the number of deaths and cases are good predictors of abnormal stock prices in the tourism sector.

